# Factors that help injecting drug users to access and benefit from services: A qualitative study

**DOI:** 10.1186/1747-597X-2-31

**Published:** 2007-10-30

**Authors:** Joanne Neale, Laura sheard, Charlotte NE Tompkins

**Affiliations:** 1 Professor of Public Health, Oxford Brookes University, Oxford, UK; 2 Research Fellow, Leeds West Primary Care Trust, Leeds, UK

## Abstract

**Background:**

International research shows that injecting drug users (IDUs) can encounter many barriers when they try to access drug treatment and other services. However, the existing literature is mostly quantitative and does not consider the kinds of factors that injectors themselves identify as enabling them to access and benefit from services. Responding to this gap in knowledge, our paper explores IDUs' own suggestions for improving service engagement and their reports of other factors enabling them to seek help.

**Methods:**

Semi-structured qualitative interviews were conducted with 75 current illicit drug injectors in three geographically diverse areas of West Yorkshire, England. Recruitment was through needle exchange programmes, with additional snowball sampling to ensure inclusivity of gender, ethnicity and primary drug injected. Transcribed data were analysed thematically using Framework.

**Results:**

Although participants were often satisfied with current access to services, they made three broad suggestions for improving engagement. These were: providing more services (more providers and more forms of support); better operation of existing services (including better communication systems and more flexibility around individual needs); and staffing-related improvements (particularly, less judgemental and more understanding staff attitudes). Other factors identified as important enablers of help seeking were: having supporting relationships (particularly with family members); personal circumstances/life events (especially becoming a parent); and an injector's state of mind (such as feeling motivated and positive).

**Conclusion:**

A range of practical suggestions for improving IDUs' access to drug treatment and other services are identified.

## Background

International research has repeatedly shown that injecting drug users (IDUs) can encounter many problems when they try to access drug treatment and other services. These problems – known as 'barriers' – relate both to aspects of service provision and its delivery (structural factors) and to the characteristics of drug users themselves (individual factors). A recent review of this research [1] noted that the published literature was largely North American and based on quantitative data. This review also suggested that barriers relating to individual factors divided into two further subcategories: firstly, injectors' demographic characteristics and personal circumstances; secondly, their psychological states of mind and treatment expectations.

The main structural barrier to help seeking is insufficient service provision – that is, the absolute lack of services for injectors [2-4] or the fact that there are not enough services to deal with drug user high demand [2,5,6]. Related to this is poor information about treatment availability, meaning that injectors do not always know about the full range of provision available to them [7,8]. Additional service-related barriers include bureaucratic hurdles, such as too much 'red tape' [9]; long waiting lists [5,10]; limited opening hours [3,11]; lack of childcare [9,12]; and stigmatising, negative or unsympathetic staff attitudes [5,13,14]. These factors can result in those who both need and want support losing their motivation.

In terms of individual barriers relating to injectors' demographic characteristics and personal circumstances, research has shown that women [5,8], members of some black and minority ethnic groups [15], homeless people [6], prisoners [16] and those who live in a rural area [6,16] can face particular problems when trying to secure support. Drug-using parents can also be reluctant to engage with services because they do not want to be separated from their children [5] or fear losing custody of them if their ability to care is questioned [14,17]. Additionally, injectors' generally chaotic and hectic lifestyles can make attending services problematic, especially when strict appointment times operate [2,18].

Individual barriers relating to injectors' psychological states of mind and treatment expectations are equally wide-ranging. Some injectors do not feel that their drug use is a problem and so do not want to seek help [7,14]. Others are too ashamed, embarrassed or guilty about their drug taking to approach professionals [9,17,19]. Some injectors are also too anxious to seek assistance in case they fail treatment [8]; their confidentiality is not respected [20]; or they discover that they are HIV positive [17]. Other injectors do not access services because they believe that the treatment available is not appropriate for their problems [21]; do not like the support on offer [10]; or assume that they are ineligible for help [4].

The existing literature thus provides much useful information on the problems IDUs encounter in accessing services. However, the focus on barriers inevitably paints a negative picture and only reveals what 'prevents' individuals from securing support. From this, policy makers and practitioners can 'infer' what needs to be done to improve user engagement. However, their inferences may not concur with what injectors themselves deem necessary and/or important. This paper seeks to fill a current gap in knowledge by exploring factors that a demographically diverse group of injectors themselves identify as 'enabling' them to access and benefit from drug treatment and other services. The term 'treatment' is used in the broadest sense of any intervention (medical or non-medical) that might modify the extent, as well as the health and social harms, of injecting drug use. The term 'services' includes specialist drug services, needle exchanges, general practitioners, hospitals, pharmacies, social services, housing services, and agencies offering support with training and education.

## Methods

Qualitative data were collected as part of a larger study of treatment barriers conducted in three geographical locations in West Yorkshire, England [1]. These three areas were a large city, a medium-sized town, and a small town within a more rural area. The research was funded by the Department of Health and involved interviews with 75 current illicit drug injectors. Ethical approval was received from a regional National Health Service Multi-Centre Research Ethics Committee and fieldwork took place between January and May 2006. Recruitment was through three needle exchange programmes (one in each area). Additional snowball sampling was employed to ensure inclusivity of gender, ethnicity, and primary drug injected and this resulted in the recruitment of five individuals, who were all in contact with one or more drug-related services. Only those who had injected in the previous seven days and were aged 18 years or older were eligible to participate.

Prior to taking part, all potential participants were assured of the confidentiality of their responses and informed that their withdrawal from, or non-participation in, the study would not affect their future care or treatment from any service. Written consent was taken and, at the end of each interview, participants were given ~10 expenses. The interviews were conducted using a semi-structured interview schedule that covered general life circumstances; drug use; treatment history/service use; problems experienced in accessing drug treatment; changes that services could introduce to make it easier for injectors to secure help; and other factors that had previously enabled, or might in the future enable, injectors to seek support.

Interviews took between 20 and 90 minutes to complete, were audio-recorded and transcribed verbatim. The interview transcripts were then coded with the aid of the software package MAXqda2. This involved reviewing each transcript line by line and indexing segments of text to a detailed coding frame that included two codes on factors enabling service access and use: i) 'suggested service changes' and ii) 'other factors enabling support seeking'. In the analyses conducted for this paper, the text segments appended to these two 'enabler' codes were exported into separate Word files and then each was analysed thematically using Framework [22]. This involved systematically interpreting and mapping the content of each file to identify the range and nature of enablers discussed and to look for associations, patterns and explanations.

## Participants

Of the 75 injectors, 28 were recruited from the large city (hereafter referred to as 'the city'), 25 from the medium-sized town (hereafter referred to as 'the town'), and 22 from the small town within a rural setting (hereafter referred to as 'the rural area'). Often, however, participants had lived in many areas and so the experiences they reported were not confined to the geographical location from which they were recruited. Fifty-two participants were male and 23 were female. Their ages ranged from 19–48 years, with no evident clustering or patterning of ages or gender across the three sites. Nine individuals were from a Black or Minority Ethnic (BME) group; 37 were single and in no relationship; 17 were in a relationship but not living with their partner; and 18 were married or cohabiting (3 missing). Fifteen participants (8 males and 7 females) had children, but only eight (3 males and 5 females) were resident with their children at the time of interview. Eight individuals described themselves as currently homeless and only 16 had been in paid legal employment during the last six months.

Nearly all of the participants reported that they had been using illegal drugs, including Class A drugs, since their teens. Forty-eight stated that their current main injected drug was heroin, 15 reported that their current main injected drug was a stimulant (crack cocaine or amphetamines) and 12 currently injected both opiates and stimulants equally. During the six months prior to interview, 52 participants had received some form of formal structured drug treatment, with 48 reporting that they had been prescribed methadone. Only one individual had had any form of residential drug treatment in the last six months, although 50 had been in contact with a general practitioner; 24 had been to a hospital; 16 had seen a probation officer; 12 had been in prison; 10 had contacted a homeless person's agency; and 5 had seen a social worker.

## Results

Participants included many sub-groups of injector (male and female; BME and white British; urban and rural dwellers; opiate and stimulant injectors; older and younger injectors). They were keen to discuss their experiences (current and previous) of accessing a diverse range of specialist addiction and generic health and social care services. Additionally, they highlighted service improvements that would enable themselves and other injectors to obtain support more easily than was currently the case. The diversity of their experiences – combined with the fact that some interesting topics were only discussed by relatively small numbers of participants – meant that it was often not possible to identify factors that helped particular subgroups of injector or facilitated access to one type of service rather than another. Nonetheless, any clear differences between respondent groups, geographical areas or service types that emerged during our analyses are reported in the text that follows.

## Suggestions for improving treatment access

When asked how addiction and generic services could change so that they were easier to approach and use, many participants (from all three areas) reported that they had no suggestions. Some qualified this by stating that services were improving all the time and everything that could be done for injectors was already being done. In particular, participants commented that needle exchange opening hours were better than they had been previously, with some pharmacy needle exchanges (and particularly supermarket-based pharmacy needle exchanges) now operating late night and weekend opening. In addition, many stated that support for a drug problem was more easily attainable within prisons and the criminal justice system than it had been in the past.

This high level of satisfaction with access to services seemed to be underpinned by three main factors. First, some injectors were initially reluctant to be critical of services from which they still needed support – however, this reticence tended to subside once the researchers had reminded participants of the confidentiality of their responses. Second, injectors commonly stated that there were limited resources available for working with drug users and so services were constrained in terms of what they could do for them. And third, injectors often argued that no service or form of support is ever perfect and so it would be wrong to be overly critical:

"I think everything's being done what possibly can be done...I don't think that there's any more they can do." [Female, 44 years, crack cocaine injector]

"What could be done better? I don't know. They are not a bad bunch of people here [needle exchange] and they do their best. And every thing has got their little teething problems and nothing is perfect." [Male, 38 years, heroin injector]

When injectors made suggestions about how both specialist addiction and generic health and social care services could be more accessible, their responses fell into three broad categories: more service provision, better operation of existing services and staffing-related improvements.

### More service provision

According to many participants, the most obvious way of making addiction and generic services easier for injectors to access would be to provide more of them. This, they argued, would require more providers (particularly in smaller towns and rural areas where service provision was currently limited), as well as a greater range of support within individual agencies. Most consistently, participants identified a need for more substitute prescribing services – both specialist community services and general practitioners who were willing to prescribe. However, they also expressed a demand for more substance misuse counsellors and mental health counsellors; more residential detoxification and rehabilitation services (especially those that accepted children, provided some medication and were not too regimented); more needle exchanges; and more outreach services, including home visiting for those with newborn babies.

In terms of the additional kinds of support wanted within existing services, injectors mostly identified further opportunities for counselling. This included formal talking therapies (particularly in the rural area) and informal advice and information that could easily be accessed on a drop-in basis (particularly in the city). Some participants (especially those who had to attend services on a daily basis) stated that they would benefit from having free bus passes or financial assistance with travelling to agencies. Small numbers of participants also wanted more information and leaflets (on drugs, drug-related health problems and where to get help); greater assistance with housing, education, and job-seeking; more diversionary leisure activities (such as opportunities for sport); and/or complementary therapies.

In addition, some heroin injectors stated that they disliked methadone because it was highly addictive and because of its side effects. Thus, they wanted access to a broader range of prescribed medications (such as, buprenorphine, dihydrocodeine and benzodiazepines). Amphetamine injectors, meanwhile, emphasised that they would appreciate any form of substitute medication since none was currently available to them. Although there was a general belief that improved access to drug treatment in the criminal justice system was a good thing, both opiate and stimulant users who had not committed crimes felt that there should be more support for drug users who were law-abiding. In this regard, many articulated a strong sense of injustice that offenders seemed to receive treatment more quickly than non-offenders and were often entitled to free bus passes to enable them to travel to their appointments:

"I think there should be more help for people who don't want to be a criminal. That is my biggest gripe of everything. I feel like the badder you are, the more help you get. If you have just got a heroin problem, the least help you get". [Female, 36 years, heroin injector]

"You shouldn't have to commit a crime to get a bus ticket because basically that is what they are doing, isn't it? They have committed a crime and they are getting the bus ticket, they are getting the medication...I think it is diabolical" [Female, 20 years, heroin injector]

### Better operation of existing services

Participants also discussed ways that services could alter their current operating procedures to become more accessible to injectors. Nonetheless, they often recognised that such changes would be difficult to implement without additional resources and/or more service provision. For example, participants from all three areas stated that waiting lists for prescribed medication should be shorter, but appreciated that this would require more prescribers. A small number of individuals wanted services (particularly pharmacies) to have private rooms, booths or sound-proofing to preserve their confidentiality and privacy. Additionally, some injectors (especially from the rural area) felt that needle exchange services needed to be open for longer, and preferably 24 hours a day. Since injectors require access to clean injecting equipment at all times of the day and night, participants were concerned that the closure of exchanges during the evenings and at weekends increased the likelihood of sharing.

"It would be better if it [needle exchange] was open when the drop-in's open on a Sunday or a Saturday. Just have a few hours on a Saturday or Sunday... On a weekend, people are struggling with needles and using other people's and that...so people who need 'em at weekend can get hold of 'em...rather than having to share." [Male, 33 years, heroin injector]

Injectors simultaneously suggested ways that services could improve their current working methods without large cost implications. For example, a small number of individuals argued that waiting times for substitute prescriptions might be reduced if some of the unnecessary bureaucracy, such as workers at drug services repeatedly asking the same questions, was removed. For example, one male heroin injector complained that being made to wait for methadone and being constantly asked the same questions seemed like methods of punishing drug users:

"I just think there is too much punishment element involved in the whole idea of providing services for you. The time that you are made to wait, especially. I know that there has to be contact with doctors and things like that but, you know, at the moment I am seeing this guy every two weeks...I am just keeping him in a job...he is just sitting asking the same old daft questions every week, week in week out. And I think it's a waste of resources, a waste of money with all these interviews that you have to go to all the time." [Male, 35 years, heroin injector]

Other participants who had been in prison felt that, despite recent improvements in drug treatment within the criminal justice system, there was still a need for better co-ordination of services for drug users when they were released. This was because individuals who had been successfully stabilised on a treatment programme in jail were often unable to continue their treatment in the community. This was either because they could not access the Drug Interventions Programme, which was a key part of the UK Government's strategy for tackling drugs and reducing crime which was introduced in 2003 or they could not find a general practitioner who was willing to continue to prescribe their substitute medication.

In addition, several injectors who had experienced long periods of waiting for community drug treatment stated that it would be helpful to be given realistic information on waiting times and a clear appointment date so that they could plan and focus, even if the overall wait for treatment could not be reduced. The period of waiting was a time of great uncertainty and instability during which many individuals reported using drugs chaotically. Knowing exactly when they would be seen by an agency could help to reduce some of the anxiety and possibly help individuals to keep their drug use under control. As this Bangladeshi injector explained:

"It [waiting for an appointment] just does your head in...It just messes around with people's head. I mean, put a date on it – yes, fair enough – you have to get them [injectors] prepared to go on it [substitute medication]...but while they are waiting to go on it, they are using. Whereas if they can set a date – 'Oh I am going to go on medication that day so I am going to be clean' – you know, that is welcome". [Male, 26 years, heroin injector]

Individuals from the rural area, where the only needle exchange service was understaffed and operated irregular opening times, also argued that needle exchange services should always try to open at the same times each day and avoid unscheduled closures, even if they could not offer longer opening hours. Their reasoning was that injectors tend not to remember complex arrangements and may resort to unsafe injecting practices if they find an exchange unexpectedly closed.

Being more accommodating of individual injectors' needs and circumstances was additionally suggested as an organisational change that could help to increase service engagement. To this end, injectors sometimes argued that pharmacies could be more flexible about when substitute medication could be collected. It was noted that some pharmacies insisted that drug-using customers could only attend between certain hours (despite being open to other customers for longer) and this created problems for those who were working, had to travel a long distance, were unwell, or had many other commitments elsewhere (for example, at housing, criminal justice or social welfare services). Other injectors felt that agencies (particularly housing services) could be more accommodating of heroin users' needs by not giving them early morning appointments. Those who use heroin, they argued, are less likely to attend services first thing in the morning because they will be experiencing opiate withdrawal and so preoccupied with obtaining either illegal drugs or their methadone prescription from a pharmacy.

Two participants also stated that they wanted drug services to be more willing to treat couples together so that they can support each other and so that an individual in treatment is not tempted to relapse because of the continued drug use of their out-of-treatment partner:

"We [self and partner] do everything together...It's important that we can do stuff together and that. Basically he has got no family and I have got no family, so basically we are both each other's support." [Female, 34 years, heroin injector]

Finally, one participant felt that waiting room arrangements in some drug services should be reviewed so that injectors are not left together in an environment where discussing drugs, dealing drugs and even threatening behaviour can occur:

"You try and keep away from doing it [taking drugs], you know, and being associated with the people who do it. But when you go down there [drug service], if you are waiting about, the more people come in and out, and it might click in your head. You might not be thinking about it [taking drugs] and they [others in the waiting room] are on about it, "Oh so and so has got some good stuff cheap"... It might put something in your head." [Male, 29 years, amphetamine injector]

### Staffing-related improvements

A number of injectors (particularly in the rural area) stated that the under-resourcing of drug services caused high staff turnover and/or understaffing which detracted from the quality of treatment that could be provided. Despite this, participants from all three areas identified improvements to the staffing of drug and other services that they felt could increase service usage without necessarily increasing costs. In particular, many injectors noted that professionals (from both specialist and generic services, as well as prisons) should be less judgemental of injectors, more welcoming of them, more understanding of their problems and more encouraging when they do make progress. Participants felt that such changes would collectively help to reduce some of the stigma, shame and embarrassment that injectors commonly experience when they first contact agencies and might encourage those with drug problems to retain contact with professionals for longer:

"The people providing these services need to...it must be infuriating...but that's just the nature of the work... Just get a bit more accepting about the fact that, you know, somebody that has come to you in the first place is at least making a start in sorting themselves out... I think that the people [drug users] could be encouraged more." [Male, 35 years, heroin injector]

In addition, injectors frequently argued that having better trained staff and more former drug users working in drug services would increase treatment uptake. Although some argued that ex-drug users can moralise or preach to those still using drugs, others felt that those who had been addicted themselves were more understanding of injectors' problems and needs and were easier to talk to than those whose knowledge was only from textbooks:

"Get some people who have been on heroin because you can like communicate more with them, do you know what I mean? And then they can tell you what to do and that because they have been there and done it. Or even people with more understanding who have researched more into it instead of just opening a book, reading a bit about it, and then going and trying to tell a heroin addict what it's like." [Male, 28 years, heroin and crack cocaine injector]

## Other factors enabling service access

When participants discussed other factors that had enabled or would enable them or other injectors to seek treatment, responses again divided into three broad categories: having supporting relationships, personal circumstances/life events and an injector's state of mind.

### Supporting relationships

Although a few participants reported that they had deliberately hidden their addiction from family members (because they were ashamed or embarrassed, did not want to upset their family or were afraid of relatives' reactions), many more (both males and females) emphasised how important family had been in enabling them to seek and obtain help. Mothers were identified as the most common providers of such support, but siblings, fathers and grandparents were also discussed. The kinds of assistance provided by family included both emotional and practical forms of help.

In terms of emotional support, injectors from all three areas reported that having someone to whom they could talk or who would listen to their problems often provided them with the confidence and encouragement they needed to seek drug treatment. In this regard, injectors reflected on how lucky they were to have family members who genuinely cared for and about them. Having relatives who trusted them and believed that they could overcome their addiction likewise helped injectors to maintain their resolve not to use drugs, especially when they were feeling low or going through a particularly difficult time:

"I can't do owt [anything] without me family really....She [mother] is giving more support. It doesn't matter even if I have lapsed, I tell her. I am very honest with her." [Female, 36 years, heroin injector]

Wanting to win back the trust of family members or aspiring to make their relatives proud were further important incentives for getting help. In contrast, those who were not emotionally supported by their family could feel that there was very little point in trying to address their drug taking:

"You do need support, know what I mean? If you haven't got no support, there's no point doing nowt [nothing]...Basically you think 'well if no-one gives a toss, why should I?"' [Male 32 years, heroin and crack cocaine injector]

The types of practical support that family members provided to injectors included phoning drug agencies to arrange treatment appointments and accompanying them to both addiction and generic services. Because injectors often lacked confidence, were desperate for treatment or struggled to express their needs clearly and calmly to professionals, having a relative who would contact a service and negotiate on their behalf was an important enabler. Physically accompanying injectors to services, meanwhile, had two clear benefits. First, it helped to ease drug users' anxieties about treatment and thus meant that they were less likely not to attend at the last minute. Second, it helped overcome transport problems by making the journey easier and cheaper. For example, this woman who lived in the town emphasized that she would not have been able to obtain methadone if her mother had not taken her to the drug service every day by car:

"It [drug service] is one of them places that it takes you so long to get in... I mean, I were quite lucky but I had to go every day for three week before I got it [methadone] and the only reason I went every day was because me mum took me. If me mum wouldn't have took me, there is no way I could have made it." [Female, 37 years, heroin injector]

Other forms of practical support that injectors received from relatives were money (for food and clothes, but also for drugs to discourage them from committing crime); temporary or permanent accommodation; meals; and child care. Although these forms of practical support did not necessarily prompt treatment seeking directly, participants acknowledged that this supportive backdrop often gave them the stability they needed to begin to address their drug use and other related problems.

In addition, injectors (again both males and females) emphasized the importance of the assistance they received from friends, peers, neighbours and partners. For example, drug-using friends and associates sometimes provided essential information about the availability and nature of local drug services and some injectors embarked on treatment and/or detoxification with friends or partners as a form of mutual support. As this woman explained:

"People say 'Oh aren't you going to so and so?' 'Haven't you heard of so and so?' And I go, 'No I didn't know that'. I didn't know that they [outreach workers] would bring needles out to you, and things like that. You just hear people talking." [Female, 42 years, heroin injector]

Injectors (particularly those from the city) also named individual drug agency workers and other professionals, such as general practitioners and pharmacists, who had made seeking assistance easier for them in the past. The reasons why these individuals had been particularly helpful related to their personal characteristics and methods of working. For example, injectors reported that professionals who were friendly, approachable, understanding and honest had put them at their ease and this had enabled them to open up. Others stated that they found it easy to seek help from drug workers who were flexible – rather than rigid in their working style – and who were readily accessible for a chat. Some injectors particularly praised professionals for the extra effort they had made in keeping them engaged with services when their motivation was low:

"I had a drug worker... and she helped me as much as she could... She was there for me all the time...I could go down and see her anytime I wanted. She came to the house and visited me...She used to phone me everyday, proper giving me help." [Male, 45 years, heroin and amphetamine injector]

As with family members, injectors reported that some drug workers had enabled them to access support by accompanying them to appointments at other services, acting as advocates for them in their dealings with other professionals, and helping them to build up their confidence. Equally, a small number of injectors stated that having drug workers and probation officers who were willing to assist them with a wide range of needs above and beyond their drug taking, such as securing accommodation or employment, had encouraged them to engage with services.

### Personal circumstances and life events

Injectors from all areas noted how personal circumstances and major life events had been instrumental in their efforts to seek help, particularly for their drug use. One of the most important of these – emphasised by both male and female participants – was being a parent, and especially becoming a parent. Key aspects of being a parent that prompted individuals to secure support related to the responsibilities of having children and needing to care and look after them – specifically, wanting to make sure that children were not taken into local authority care or getting children back from care; wanting to see more of their children; not wanting their children to know that they used drugs; and being tired of having little money to spend on children.

Other life events that prompted help seeking were bereavements (relating to parents, friends and partners), family illnesses, periods of poor mental health, and in one case having a leg amputated. Sometimes, individuals realised that they needed professional help because they were simply not coping with what had happened. Sometimes the events heightened a sense of concern about their poor health and/or fear of inflicting permanent damage on themselves through injecting:

"Only reason that I really decided I wanted help was fact that me injecting sites were getting bad and I ended up injecting in groin... Just couldn't find a vein to inject in and ended up trying me groin and ultimately people's legs were dropping off and I thought, 'I don't want me leg to drop off... It's not that good"'. [Male, 37 years, heroin injector]

Other participants reported that professionals seemed to be more sympathetic and willing to help them when they were having personal difficulties. For example, one man reported that drug service staff had only prescribed him methadone because his mother was very ill and another described how he had been fast-tracked for methadone after attempting suicide. A third explained how the death of his mother, combined with his own health problems, had led to him being able to stay in treatment despite the fact that his doctor had wanted to expel him for obtaining illicit prescriptions:

"Family friends took me down and they sat down with me doctors and these other people and they [the professionals] said, 'If it wasn't for your situation...we would kick you out [of treatment] and you wouldn't be getting anything."' [Male, 29 years, heroin and crack cocaine injector]

Further personal circumstances that participants perceived as facilitating help seeking were having transport (particularly in the town and rural area) as this made it easier to attend appointments; being vulnerable (particularly being a young female, having small children, or having experienced domestic violence) which increased an individual's priority for drug treatment and housing; being in the criminal justice system which seemed to result in fast access to prescribed substitute drugs; and moving into a new home which afforded sufficient stability to begin to address drug and other problems.

### An injector's state of mind

Lastly, some injectors (mostly male) explained how feeling more motivated and positive about themselves had been a factor in prompting them to seek help. These individuals commonly emphasized that there was no point in entering drug treatment until they were genuinely motivated to address their drug taking – otherwise any treatment received would fail. The kinds of factors that seemed relevant to increased motivation were feeling less depressed about their lives than previously, a growth in self-confidence and will-power, and reduced feelings of shame and embarrassment about themselves and their behaviour.

Precise reasons for these positive emotional and psychological changes were not always easy to identify, although having a supportive drug worker, getting married, and becoming a parent were all discussed. It was also the case that participants who reported better psychological and emotional health tended to be those who had already had a treatment encounter that had left them feeling 'helped' even if not 'cured':

"I used to think if I asked someone for help, they would see me as inadequate or er...not up to their level. I don't know. And I wouldn't ask for help...Because I thought they would see me as different... Then I did an enhanced thinking class in prison...It just showed me how to think different. Look at long-term things... After that I just thought, 'If you don't ask for help, you are not going to get any help"'. [Male, 33 years, heroin injector]

Significantly, however, those who described the kinds of psychological and emotional improvements that pre-empted help-seeking tended to be those who had also made a firm decision that they wanted to be drug free. Some reported that they were tired of using drugs, disliked having a life that was complicated or ruled by addiction, wanted to stop committing crime, or were afraid of dying. Others recognized that other things (such as being a parent, going to college, getting a job, or owning a house) were more important than drug taking.

## Discussion

Despite widespread international evidence that drug treatment is effective in reducing levels of drug taking, criminal activity, health risks, and the sharing of injecting equipment [23-28], previous research has shown that IDUs encounter many problems when they try to access specialist addiction and more generic services. Responding to this, the UK National Treatment Agency (NTA) has committed itself to ensuring that drug treatment is available promptly to all who need it and stipulated that all drug treatment services should be easily accessible by virtue of location, entry criteria, assessment procedures, and waiting times [29].

Thus, there is a clear will at the UK policy level to improve IDUs' access to services. However, understanding how best to do this is currently constrained by a number of factors. First, most of the research on treatment barriers has been conducted in North America [1]. We cannot assume that research undertaken in other countries – where patterns of drug use, policies and service provision are different – will necessarily apply to the UK setting. Moreover, the ever-changing nature of drug-taking behaviours and service delivery suggests that the problems of accessing services will vary over time, as well as place. Consequently, findings on treatment barriers that applied in the past may not necessarily apply currently or in the future.

Second, most previous research has been quantitative rather than qualitative in design [1]. Quantitative research is well-suited to revealing which types of drug users are likely to experience which kinds of barriers. Nonetheless, both the types of barrier considered for inclusion in such studies and the types of strategy suggested for responding to the barriers reported tend to be determined by researchers and policy makers rather than by drug users themselves. Without more service user input, current understanding of what is important in improving access is likely to be one-sided and incomplete. Third, factors that hinder service use will not necessarily be the mirror opposites of factors that enable drug users to access support. Undoubtedly, there will be overlap between the two phenomena. Yet, researching factors that help injecting drug users to access and benefit from services could highlight issues about engagement that would be missed in a study focused on barriers alone.

In order better to inform UK policy and practice, the present paper sought to add to the existing literature on IDUs' access to services by allowing injectors from West Yorkshire, England, to identify and discuss factors that they personally thought enabled them to secure the help they needed. Inevitably, there are limitations with the data presented. For example, qualitative research seeks in-depth information from relatively small samples, so reducing representativeness in order to maximise validity. Caution should therefore be taken in generalizing the findings to other locations, even within the UK. In addition, participants were mainly recruited through needle exchange programmes. The views and experiences of individuals not using needle exchange services – perhaps because they wanted to keep their drug use hidden or because they did not know that such services existed – are therefore not included in the analyses.

Additionally, it was mostly not possible to identify robust differences between the views and experiences of the various sub-groups of injectors participating in the research. This was because injectors often belonged to more than one subgroup, drew on both their current and previous treatment-seeking experiences, and reflected on others' needs as well as their own. Furthermore, some of the factors identified as enabling access were only reported by relatively small numbers of participants. These issues were often very insightful (and others might have concurred with their relevance had they been explicitly asked). Nonetheless, the small numbers of individuals spontaneously discussing them prevented us from ascertaining whether or not there were meaningful patterns in the responses based on injectors' demographic or drug use characteristics.

In spite of these shortcomings, the data have important strengths. The informal atmosphere of the semi-structured interview, and the complete independence of the research team from any service provider, enabled participants to be open and reflective about their experiences and minimised the danger of them over-emphasising the positives of service access and under-playing the barriers. Also, by allowing participants to talk freely – rather than asking them predefined questions with fixed responses – the study produced thoughtful and detailed accounts that could be analysed inductively by searching for emergent themes and patterns [30]. Finally, by sampling as inclusively as possible, our findings successfully captured a very wide range of factors enabling service access, incorporating both common and more esoteric issues.

A key finding that distinguished the present research from previous studies was our participants' evident appreciation of current service access and their frequent inability to suggest ways that services could improve. This was an unanticipated and positive recurrent theme, but one which required further scrutiny. For example, it was possible that injectors were – despite the researchers' reassurances of confidentiality – still anxious about being critical of services or found the questioning about suggested changes too vague. Although plausible, such arguments should perhaps not be overstated given that the topic of improvements was not introduced until late in the interview, and therefore after considerable discussion of the problems of accessing treatment had already taken place. Furthermore, injectors related their satisfaction to three clear issues: improvements in service provision that they believed had taken place in recent years, their feelings regarding what services might realistically offer, and the fact that all services have problems.

These findings suggested that injectors' satisfaction tended to be genuine, but related at least in part to their modest expectations about both service accessibility and their entitlement to treatment. Indeed, accessibility was far from perfect and many injectors identified the need for more services and/or improvements to existing provision. Our findings relating to the need for more services were consistent with the existing treatment barriers literature, but produced further detail on the wide range of additional support desired (such as substitute prescribing, counselling, residential services, needle exchanges, outreach, advice and information). Equally, we uncovered some further information on which subgroups of injector were requesting particular types of help. For example, those in the rural areas wanted more formal counselling; those in the city identified a need for informal advice and information that could easily be accessed on a drop-in basis; those who had to attend services on a daily basis wanted financial help with travelling to agencies; and amphetamine users desired substitute medication.

Other suggested changes to existing services included better operating procedures and staffing-related improvements. Again, there were strong parallels between the participants' reports and previous international research on treatment barriers. For example, suggested changes included shorter waiting lists; longer opening hours; less bureaucracy; more flexibility around appointments; and less negative and judgemental attitudes. Nonetheless, our focus on enablers – rather than barriers – indicated that information on realistic waiting times and a clear appointment date would be helpful even if the overall wait could not be reduced. Similarly, regular opening times could prove valuable, even if longer operating hours were not possible. IDUs also explained that those who were stabilised on substitute medication whilst in prison could not always find someone to continue their prescription on release. Thus, they advocated better co-ordination of services for ex-prisoners. In addition, two individuals wanted a greater willingness to treat drug-using couples together and one felt that waiting room arrangements should be reviewed.

Whilst negative staff attitudes have already been documented as a deterrent to service use within the existing barriers literature, less information has been provided on 'how' staff attitudes might be changed to facilitate help seeking. Our data revealed that IDUs wanted professionals to be more welcoming, understanding of their problems and encouraging of their efforts. They also desired staff who were knowledgeable about drug issues, including former drug users who understood the problems they faced. Such findings corroborate other research on the key characteristics of effective addiction practitioners. That is, they should genuinely respect their clients, convey appropriate levels of empathy, comprehend their situations – including the effects that substance misuse has had on them and on those around them – and understand what needs to be done in response to their problems [31,32].

The importance of having supportive people to assist when help seeking was another key finding of our research that has not been fully explored in previous studies of treatment barriers. This is despite good evidence from the broader addictions literature that substance misuse does not affect individuals in isolation from their social networks and involving family members and friends in treatment processes helps to promote positive treatment outcomes [33,34]. Our analyses thus add to the literature on service access by highlighting that barriers do not simply occur at the individual and service level. Rather, they can also be a feature of injectors' poor family relationships and limited social networks.

Finally, our data on IDUs' other personal, social and psychological characteristics reinforce the importance of client motivation and feelings of shame and embarrassment in help seeking. In addition, they indicate how both positive and negative life events can act as triggers to accessing support. Again, there is already a wealth of literature showing how motivation, "hitting bottom", qualitatively changed life perspectives, and positive and negative life experiences can all prompt individuals to address their drug use [35-38]. Despite this, the existing literature on treatment barriers has not tended to emphasise that access to services might be improved by promoting injectors' self-esteem, confidence and motivation; and by targeting offers of support at times of significant life change. By focusing on factors that enable rather then impede service use, our study has brought this to the fore.

## Conclusion

In conclusion, some policy and practice implications arising from our research are reviewed. Like our injectors, we appreciate that resources are finite and no service is ever perfect. Thus, we refrain from compiling a wish list that simply indicates an unrealistic need for more of everything. Moreover, in keeping with our philosophy of learning directly from what our participants themselves said, we do not stray too far from the points they personally made. Consequently, some of our suggestions will be relevant to many injectors across a wide range of services – including those located beyond West Yorkshire. Others will reflect the needs and wishes of relatively few and/or be pertinent to West Yorkshire alone. Inevitably, service commissioners, providers and workers will have to reflect on the points raised within the context of their own locality and client mix. However, our findings suggest that injectors are likely to both notice and appreciate any positive changes made.

To begin, we recommend that the current UK policy commitment to improve drug treatment access should be continued and increased. In particular, shorter waiting lists for substitute medication appear to be desired. However, if these are not possible, realistic information on waiting times and a clear appointment date would help. Official waiting list data do not always provide the most accurate indicator of how long it will take for an individual to receive treatment [39]. Nonetheless, at the time of our data collection, figures published by the National Treatment Agency indicated that the national average waiting time for specialist prescribing was 2.8 weeks with a target of 3 weeks. Published data on waiting periods for specialist prescribing in the study areas were 4.1 weeks in the city, 0.9 weeks in the town and 2.8 weeks in the rural area [40]. This suggested that injectors from the city were likely to have been waiting longer, and those in the town waiting less, than many other injectors nationally.

Our participants also identified a need for longer operating hours at needle exchange services, and regular opening hours even if these were not possible. Although this was mostly stated in respect of the rural area, the desire for 24-hour opening was evident in all three study locations. In practice, 24-hour access to needle exchange facilities is uncommon, most likely due to the costs that would be involved. Nonetheless, needle vending machines – as already operate in a number of other countries – might be considered as a cheaper and more feasible alternative. In terms of reducing unnecessary bureaucracy, better formal and informal communication systems within individual services (such as workers routinely passing verbal and written reports of information collected from clients to other workers), improved joint working between agencies (particularly community and criminal justice services), and the sensitive use of computerised record keeping (subject to confidentiality and data protection requirements) could all help to reduce the number of times individuals are asked to repeat information about themselves. Additionally, agencies should regularly review the exact information they need from new clients and omit any information that is extraneous.

For those whose lives are particularly chaotic, those who are repeatedly in contact with a wide range of different agencies, and those who have to travel long distances to reach services, greater flexibility around appointments should be contemplated. Specifically, pharmacies could cease placing restrictions on the times when drug users are permitted to collect their methadone and other services could try to avoid giving known drug users early morning appointments, thus minimising the likelihood that they will be in withdrawal whilst waiting to be seen. Drug services might also expand their use of drop-in sessions and, as part of a broader drive to meet clients' diverse needs, could consider accepting couples into treatment programmes together. The latter might help some to sustain their motivation and prevent a drug-using partner hampering the efforts of an individual who is desperate to be drug-free.

The provision of help with travel to services – in the form of bus fares or bus passes – could prove difficult to administer and may not necessarily ensure attendance. However, it should not be a particularly expensive intervention and could usefully be assessed on an individual basis. Indeed, support with travel is now provided by a range of services elsewhere in the UK, thus suggesting that this could have been implemented more widely within the study area. Arguably, this would have helped to dispel the participants' perception that those who were law-abiding were treated less favourably than those within the criminal justice system because they had to pay for their own travel whilst offenders travelled free. More fundamentally, the improvements in access to treatment within the UK criminal justice system that have been evident in recent years must now be matched by improvements within the community. This would help to ensure that prisoners do not lose the support that they have been receiving in prison once they are released.

In addition, efforts must be made to reduce the negative attitudes drug injectors repeatedly report from their contact with a wide range of service providers. In this regard, our participants highlighted the need for more welcoming, understanding and encouraging professionals, particularly those who were knowledgeable about drugs. Such findings have implications for staff training and education. For example, it seems reasonable to assume that professionals are more likely to be sympathetic to drug users if they comprehend the often complex nature of their problems and the negative impact that hostility and stigma can have on treatment processes [13,41]. This increased understanding might be achieved through formal education on drug-related issues, but also through more interagency working whereby specialist addiction workers collaborate with generic professionals, thus passing on some of their relevant knowledge and skills.

According to our participants, the employment of former drug users in drug services could further help to reduce stigma and unfound prejudice. We recognise that this might be complex. Ex-users may relapse and some activities require specialist knowledge that may not be easily acquired. Indeed, some of our participants themselves reported that former drug users have a tendency to moralise or preach. Nonetheless, individual services should carefully consider the kinds of functions that both current and ex-users might play in the delivery of support to other clients. For example, those who have themselves been through the drug treatment system could act as supportive peers and mentors to those who are anxious about attending services, lacking in confidence, or without other forms of informal support. Service providers should thus develop and champion forms of peer-led support, user involvement and volunteer programmes whenever possible. Indeed, the involvement of service users in planning, commissioning and delivering drug treatment services is already being implemented in many areas of the UK with research and practice guidance now illustrating how best to take this forwards [42-44].

Lastly, our findings suggested that engagement with services might be increased by encouraging supportive relationships between IDUs and members of their non-drug using social networks and targeting offers of support at times of significant life change. Such strategies already underpin a range of specific approaches to addiction – for example Systemic Family Intervention [45], the Strengthening Families Program [46] and Motivational Enhancement Therapy [47]. However, they could be adopted more routinely by a wider range of professionals. Equally, it might be possible for service providers to increase user engagement by simply taking more time to learn about their clients' lives and families. Such information could enable them better to understand when interventions might be most appropriate, who else might offer support, and what support those individuals might themselves need.

## Competing interests

The author(s) declare that they have no competing interests.

## Authors' contributions

JN played a key role in designing the study and led on the analyses and writing of the paper. CT and LS interviewed participants and collected all data for the study. They both contributed to the analyses, and were involved in writing and redrafting the paper. All authors read and approved the final manuscript.
